# Racial disparity and prognosis in patients with mouth and oropharynx cancer in Brazil

**DOI:** 10.4317/medoral.25334

**Published:** 2022-04-03

**Authors:** Letícia Francine Silva Ramos, Adriano Referino da Silva Sobrinho, Lucas Nascimento Ribeiro, Allan Vinícius Martins-de-Barros, Herika Arruda Maurício, Stefânia Jeronimo Ferreira, Marianne de Vasconcelos Carvalho

**Affiliations:** 1 Undergraduate in dentistry. University of Pernambuco, Arcoverde, Brazil; 2 Degree in dentistry. University of Pernambuco, Arcoverde, Brazil; 3 Postgraduate program in dentistry. University of Pernambuco, Camaragibe, Brazil; 4 Academic Doctorate in Public Health. Aggeu Magalhães Institute, Brazil; 5 Adjunct Professor of Dentistry. University of Pernambuco, Arcoverde, Brazil; 6 Vice-Coordinator of the Postgraduate Program in Dentistry. University of Pernambuco, Camaragibe, Brazil

## Abstract

**Background:**

Oral and oropharyngeal cancer (OPC) is an important cause of morbidity and mortality worldwide. Populations in situations of social vulnerability tend to have higher incidences of cancer, a higher proportion of late diagnosis, greater difficulties in accessing health services, and, consequently, worse prognosis. The aim of this study was to evaluate the relationship between race/skin color and OPC prognosis in Brazil.

**Material and Methods:**

This is a cross-sectional epidemiological study using OPC data from the National Cancer Institute between the years 2000 and 2019. The selected variables were: gender, race/skin color, age, education, smoking and alcohol consumption, stage of the disease and disease status at the end of the 1st treatment.

**Results:**

154,214 cases were recorded. Black men, in the 6th decade of life, were the most affected population. Blacks had a lower level of education when compared to non-blacks (*p*<0.001). Blacks were more exposed to smoking and alcohol consumption (*p*<0.001). At the time of diagnosis, the black population was at the most advanced stage when compared to non-blacks (*p*<0.001). At the end of the 1st treatment, more black patients had disease in progression, as well as more black patients died (*p*<0.001).

**Conclusions:**

Blacks had a worse prognosis for OPC in Brazil. Despite the limitations, these results are important to elucidate the scenario of health disparities in relation to the race/skin color of the Brazilian population.

** Key words:**Head and neck, oncology, cancer, oral cavity, oropharynx.

## Introduction

Cancer is a leading cause of morbidity and mortality worldwide and differs greatly across racial and ethnic groups (1). A cohort study with cancer patients revealed that there are variations by race/skin color in the diagnosis, treatment and survival of cancer. This cohort study included 950,377 Asian, black, white, and Hispanic patients diagnosed with prostate, ovarian, breast, stomach, pancreatic, lung, liver, esophageal, or colorectal cancer. Data were collected on the basis of Surveillance, Epidemiology and End Results, and patients were observed for more than 5 years. Compared with Asian patients, blacks are more likely to have metastatic disease at diagnosis, and black and Hispanic patients were less likely to receive treatment (1).

Some of these differences may reflect specific genetic characteristics and may be related to socioeconomic and cultural factors (2,3). Populations in situations of social vulnerability tend to have higher incidences of cancer, a higher proportion of late diagnosis, greater difficulties in accessing health services, and, consequently, worse prognosis, lower survival rates and higher rates of mortality from the disease (1,4-8).

Oral and oropharyngeal cancer (OPC) is an important public health problem worldwide. Annually, more than 442,000 new cases are diagnosed worldwide (9). An association between race/skin color and lower survival rates for OPC has been observed. The black population seems to have the diagnosis made in more advanced stages (10). However, in Brazil, this scenario has been little explored. Studies relating data such as mortality, morbidity and stage of diagnosis with race/skin color are scarce. Surveillance of trends in OPC morbidity and mortality across race/skin color can contribute to health programs that reduce the burden of disease and minimize health differences that are unfair, avoidable, and unnecessary (11). Thus, the aim of this study was to evaluate the relationship between race/skin color and the prognosis of OPC.

## Material and Methods

- Ethical considerations

As it is a research that uses information available in a database in the public domain, the study did not require approval by the Research Ethics Committee (based on Resolution No. 510/16 of the National Health Council).

- Study design

This is a cross-sectional epidemiological study using secondary data extracted from the Hospital Cancer Registries (RHC) of the National Cancer Institute (INCA). This database is an instrument resulting from the reorganization of cancer care policies in Brazil and has been consolidated as a tool for epidemiological surveillance of cancer, in order to guide the development of research for the planning of disease control actions throughout the national territory.

- Data collection

Data from hospital records of cancer cases between the years 2000 and 2019 were accessed through the Integrador RHC, available for public access at https://irhc.inca.gov.br/RHCNet, which is an online tool of consolidation of databases that makes access to hospital information on cancer easy, fast and public. The RHCs have information on patients treated in hospital units with a confirmed diagnosis of cancer, and it is supplied, obligatorily, by hospitals qualified in Specialized Care in Oncology of the Unified Health System (SUS) and, optionally, by hospitals not qualified.

Cases of oral and oropharyngeal cancer were identified and filtered in the Integrator RHC through the International Codes of Diseases Version 10 (ICD-10) referring to the following anatomical locations: lips (except lip skin); lip mucosa; labial commissure; language base; tongue; gum; floor of mouth; hard palate; soft palate; uvula; cheek mucosa; mouth vestibule; retromolar area; other parts of the mouth and unspecified parts; tonsil fossa; tonsillar pillar; tonsils; and oropharynx; which correspond to the ICD-10 codes C00 to C06 and C09 to C10.

For the purpose of this study, the variables selected for the analyzes were: Sociodemographic: gender (female and male), race/skin color (black and non-black), age at diagnosis (decade of life). Variables related to risk factors: education (none, up to 12 years and higher education), smoking and alcohol consumption (user/ex-user and never). Variables in relation to prognosis: staging (I, II, III and IV) and disease status at the end of the first treatment (complete remission, partial remission, sTable disease, disease in progression, out of therapeutic possibility and death).

All variables were extracted from the information system itself and categorized according to the proposed analyses. The answer options “no information”; "not applicable"; “not evaluated” and “ignored” were recorded as losses and excluded from the analyzes.

- Database construction and statistical analysis

The collected data were tabulated in the SPSS® statistical software platform in its version 20.0, with subsequent verification of data consistency. Descriptive and inferential statistical analyzes were performed. In the descriptive analysis, the data of the variables were presented in the form of absolute and relative frequency.

For inferential statistical analysis, the variable “race/skin color” was the main independent variable of the study and was categorized into black (black and brown) and non-black (white, indigenous and yellow), according to the criteria of the Brazilian Institute of Geography and Statistics (IBGE) for aggregation by race/skin color, while the dependent variables “history of alcohol consumption” and “history of tobacco consumption” were dichotomized into 'yes' for individuals who consume or have consumed the substance and 'no' for individuals who have never consumed them.

The relationship between the independent variable “race/skin color” and the nominal categorical dependent variables related to the risk of oral and oropharyngeal cancer was evaluated using the Person chi-squared test and the calculation of prevalence ratios (PR) and their respective 95% confidence intervals, while the nonparametric Mann-Whitney test was used for the ordinal categorical dependent variables related to the prognosis of oral and oropharyngeal cancer. For all statistical tests, a significance level of 5% was adopted (*p* < 0.05).

## Results

There were 154,214 cases of oral and oropharyngeal cancer recorded in the analyzed period. The black population had most of the cases (52.70%). The male gender was responsible for 77.03% of the registered cases. At the time of diagnosis, the sixth decade of life (50-59 years) (33.85%) was the most affected, followed by the seventh decade of life (60-69 years) (27.37%) (Table 1).

Regarding the level of education, most black and non-black people (75.08%) studied until the age of 12. However, more black people (22.94%) had no level of education when compared to the non-black population (11.66%). Most patients had the habit of consuming tobacco and alcoholic beverages. The black population showed to be more related to these risk factors (Table 2).

Regarding the stage of the disease at the time of diagnosis, about 60.38% of blacks were in the most advanced stage of the disease (stage IV) compared with 53.04% of non-blacks. Complete remission of the disease at the end of the first treatment was more frequent in the non-black population (32.47%) than in the black population (22.23%). Additionally, more black patients had disease progression (16.20%), as well as more black patients died (24.12%) (Table 3).

All analyzes showed a statistically significant association, with *p*<0.001.

**Table 1:**
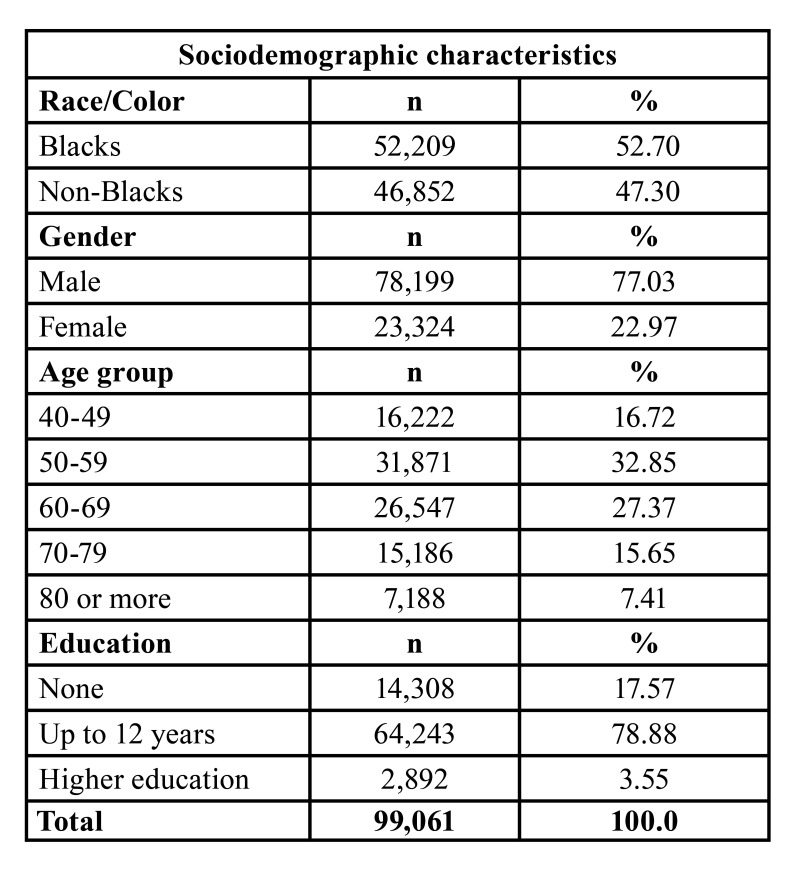
Sociodemographic characterization of oral cancer cases in Brazil between 2000 and 2019.

**Table 2:**
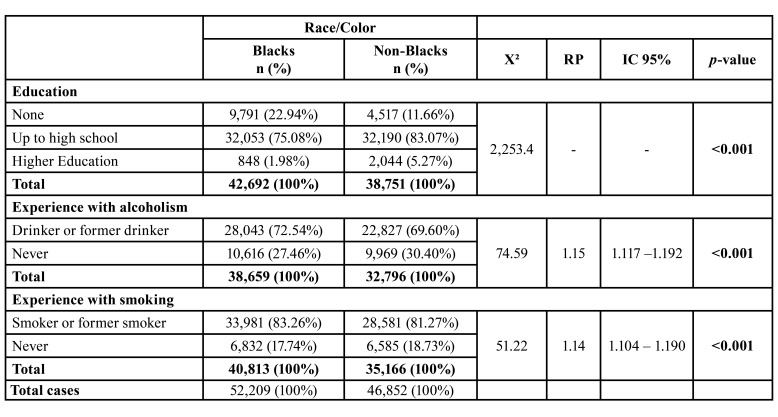
Relationship between the variable Race/Skin Color and risk factors for oral cancer.

**Table 3:**
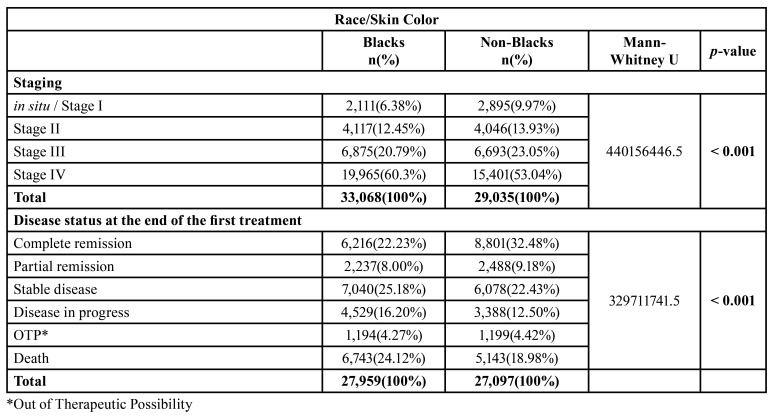
Relationship between the variable Race/Skin Color and prognostic factors for oral cancer.

## Discussion

In this study, the male gender and the age group between 50 and 59 years were more related to the development of OPC, which corroborates the profile found in the literature of patients most affected by this type of cancer (12,13). Among diagnosed patients, the black population had a higher incidence of OPC. Other research has also found higher incidences of this type of cancer among black patients (12,14).

Tobacco use and alcohol consumption are established risk factors for OPC (15). In the present study, it was identified that the black population was more exposed to these factors. The habit of smoking has been more related to blacks when compared to whites (16). Although recent studies in Brazil on the prevalence of smoking do not have cuttings by race/color, it has been shown that people with less education are more likely to smoke and have had, in recent years, a smaller decrease in the incidence of this habit compared to people with higher levels of education (17). In the present research, black patients showed the worst levels of education.

The higher incidence of OPC among black patients in this study can also be explained by the greater exposure of these patients to smoking and alcohol consumption. Awareness of the causes of OPC and the signs and symptoms are information that need to be disseminated through health education, with programs aimed mainly at high-risk groups. Once aware of the role of risk factors, primary prevention favors the reduction of these factors (18).

It has been highlighted that the race/color of patients may be an independent risk factor for mortality in cases of head and neck cancer (19). Black patients diagnosed with oropharyngeal cancer are more likely to have distant metastases and unresecTable tumors, (10) being diagnosed at more advanced stages (20). In addition, the survival rate has been reported to be lower for black patients with OPC (21).

In a study carried out in São Paulo, Brazil, between 2003 and 2009, mortality for black patients with oral cancer doubled compared to white patients (11). The results related to the poor prognosis of OPC in the present study are related not only to the risk factors mentioned, but also to socioeconomic factors, such as access to health care.

Access to the public health system proved to be more precarious among black individuals. In addition to the level of education, access to health care in this group may be impaired due to the structural racism existing in the country (22). Racism is rooted in Brazil and leads to disadvantages in access to services, either because of the lack of health units close to the locations where the black population is concentrated, or because of the scarcity of health programs aimed at this population (22). There is, therefore, a need for interventions aimed at minimizing disparities in relation to color/race in the health system (21).

As it is a study carried out with secondary data, this research has some limitations. With regard to the decrease in cases from 2017 onwards, this data may be related to the structure of the database used. The process of sending data to this database is dynamic, as health facilities are enabled and disabled over time and this can influence the number of records reported. It is also possible for hospitals to send information retrospectively, which modifies the number of records (23). Another limitation of this study was the impossibility of including the diagnosis for Human Papillomavirus (HPV), given the importance of this factor for oropharyngeal cancer. RHCs do not have this information available.

In this study, black patients were diagnosed at more advanced stages and had a worse prognosis for oral and oropharyngeal cancer in Brazil. Despite the limitations, these results are important to elucidate the scenario of health disparities in relation to the race/skin color of the patients. These findings should be further explored by future research, aiming at the development of public policies that lead to the reduction of health inequalities in the country.
